# 1-(3,4-Dimeth­oxy­phen­yl)propan-1-one

**DOI:** 10.1107/S1600536811035173

**Published:** 2011-09-14

**Authors:** Biao Yang, Changri Han, Xiaoping Song, Guangying Chen, Xinming Song

**Affiliations:** a Key Laboratory of Tropical Medicinal Plant Chemistry of the Ministry of Education, College of Chemistry & Chemical Engineering, Hainan Normal University, Haikou 571158, People’s Republic of China

## Abstract

The title compound, C_11_H_14_O_3_, was isolated from the stems of *Trigonostemon xyphophylloides*, which belongs to *Trigonostemon* genus of Euphorbiaceae. The plants in this genus were used in folk medicine, such as for the treatment of diseases caused by viruses and fungi. The limited investigation of the chemistry of this plant prompted an examination of constituents of its twigs, from which the title compound was isolated. The mol­ecule is approximately planar with an r.m.s. deviation of 0.1237Å. In the crystal, inter­molecular C—H⋯O hydrogen bonds connect the mol­ecules into a two-dimensional network structure with an *R*_2_^2^(12) graph-set motif.

## Related literature

For the medicinal and botanical background to the title compound, see: Zdero *et al.* (1990 ▶); Lopes *et al.* (1996 ▶). For weak hydrogen bonds, see: Steiner (1996 ▶).
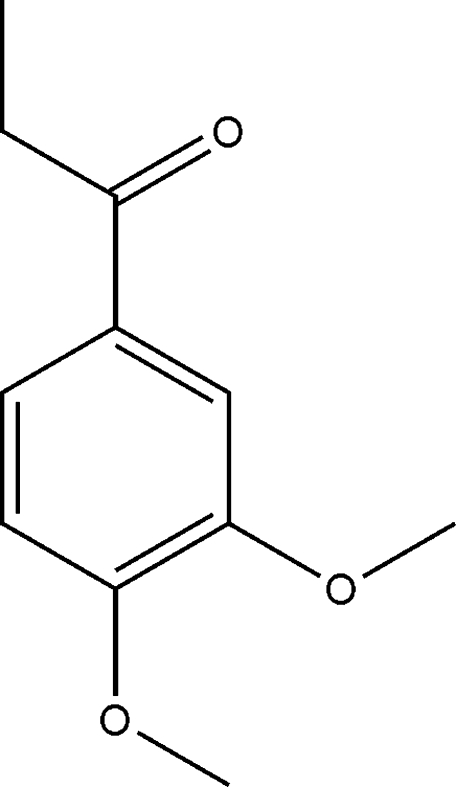


## Experimental

### 

#### Crystal data

C_11_H_14_O_3_
*M* *_r_* = 194.22
Monoclinic, 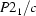

*a* = 8.9308 (9) Å
*b* = 13.8582 (14) Å
*c* = 8.5692 (8) Å
β = 102.427 (1)°
*V* = 1035.72 (18) Å^3^
*Z* = 4
Mo *K*α radiation
μ = 0.09 mm^−1^
*T* = 298 K
0.50 × 0.43 × 0.40 mm

#### Data collection

Bruker APEXII CCD diffractometer
Absorption correction: multi-scan (*SADABS*; Sheldrick, 2003 ▶) *T*_min_ = 0.236, *T*_max_ = 0.965
5082 measured reflections
1827 independent reflections
1265 reflections with *I* > 2σ(*I*)
*R*_int_ = 0.024

#### Refinement

*R*[*F* ^2^ > 2σ(*F* ^2^)] = 0.041
*wR*(*F* ^2^) = 0.122
*S* = 1.04
1827 reflections
131 parameters
H-atom parameters constrained
Δρ_max_ = 0.16 e Å^−3^
Δρ_min_ = −0.18 e Å^−3^

### 

Data collection: *APEX2* (Bruker, 2003 ▶); cell refinement: *SAINT-Plus* (Bruker, 2003 ▶); data reduction: *SAINT-Plus*; program(s) used to solve structure: *SHELXTL* (Sheldrick, 2008 ▶); program(s) used to refine structure: *SHELXTL*; molecular graphics: *SHELXTL*; software used to prepare material for publication: *SHELXTL*.

## Supplementary Material

Crystal structure: contains datablock(s) global, I. DOI: 10.1107/S1600536811035173/nr2005sup1.cif

Structure factors: contains datablock(s) I. DOI: 10.1107/S1600536811035173/nr2005Isup2.hkl

Supplementary material file. DOI: 10.1107/S1600536811035173/nr2005Isup3.cml

Additional supplementary materials:  crystallographic information; 3D view; checkCIF report

## Figures and Tables

**Table 1:** Hydrogen-bond geometry (Å, °)

*D*—H⋯*A*	*D*—H	H⋯*A*	*D*⋯*A*	*D*—H⋯*A*
C11—H11*C*⋯O2^i^	0.96	2.66	3.607 (2)	171
C8—H8⋯O1^ii^	0.93	2.50	3.419 (3)	171

Symmetry codes: (i) 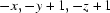; (ii) 
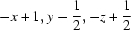
.

**Fig. 1.**
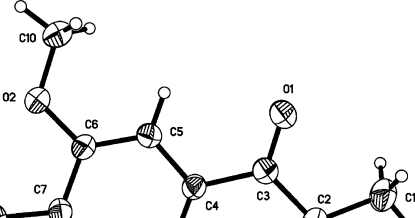
View of the title compound without the hydrogen atom-labeling scheme. Displacement ellipsoids are drawn at the 30% probability level.

**Fig. 2.**
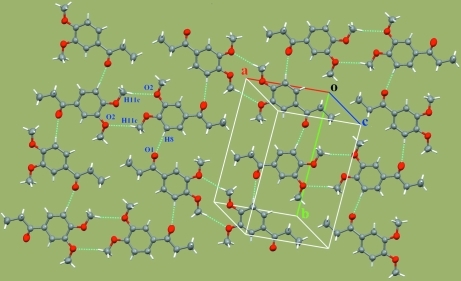
A view of the molecular packing.

## supplementary crystallographic information

### Comment

The title compound was isolated from plants such as *Pteronia camphorata* (Zdero *et al.*, 1990) and *Virola surinamensis* (Lopes *et al.*, 1996). In our ongoing studies of natural products with biological activity we isolated the compound from the 75% EtOH extract of the stems of *Trigonostemon xyphophylloides*, a plant used as a folk medicine which were collected from Jianfengling County, Hainan Province, P.R. China. We have undertaken the X-ray crystal structure analysis of the title compound in order to establish its molecular structure and relative stereochemistry.

The molecular structure of (I) is shown in Fig.1. All nonhydrogen atom are coplanar, the mean deviation is 0.1237Å  and the largest deviation being -0.3235 (20)Å for atom O1.In the crystal, molecules are linked by intermolecular C–H···O hydrogen bonds into two dimensional network structure (Steiner, 1996) (Fig.2). There are two intermolecular C–H···O hydrogen bonds C8–H8···O1 and C11*c*–H11*c*···O2, each adjacent C11*c*–H11*c*···O2 form a ring of twelve atoms with with an *R*^2^_2_(12) graphset motif.

### Experimental

Air-dried stems of *Trigonostemon xyphophylloides* (5.9 kg) were ground and percolated (3 × 2.5 h) with 75% EtOH at 60°C, which was suspended in 1.5 *L* water and then partitioned with petroleum ether, chloroform, ethyl acetate and n-BuOH, successively, yielding a petroleum ether extract, a chloroform extract, an ethyl acetate extract and a n-BuOH extract, respectively. The petroleum ether extract was subjected to a silica gel CC column using petroleum ether as first eluent and then increasing the polarity with EtOAc, to afford 20 fractions (A—T). Fraction D was further separated by column chromatography with a gradient of petroleum ether-EtOAc to give the title compound. The crude product was dissolved in small amount of ethyl acetate to obtain single crystals suitable for X-ray analysis by slow evaporation of ethyl acetate solution at 298 K.

### Refinement

H atoms were positioned geometrically and refined as riding groups, C—H = 0.93 Å for aromatic H, 0.96 Å for methyl H,0.97 Å for methylene H and constrained to ride on their parent atoms, with *U*iso(H)= *xU*eq(C), where *x* = 1.2 for aromatic H and methylene H, and *x* = 1.5 for other H.

### Crystal data

**Table:**

C_11_H_14_O_3_	*F*(000) = 416
*M**_r_* = 194.22	*D*_x_ = 1.246 Mg m^−^^3^
Monoclinic, *P*2_1_/*c*	Mo *K*α radiation, λ = 0.71073 Å
Hall symbol: -P 2ybc	Cell parameters from 1875 reflections
*a* = 8.9308 (9) Å	θ = 2.4–23.9°
*b* = 13.8582 (14) Å	µ = 0.09 mm^−^^1^
*c* = 8.5692 (8) Å	*T* = 298 K
β = 102.427 (1)°	Block, colourless
*V* = 1035.72 (18) Å^3^	0.50 × 0.43 × 0.40 mm
*Z* = 4	

### Data collection

**Table:**

Bruker APEXII CCD diffractometer	1827 independent reflections
Radiation source: fine-focus sealed tube	1265 reflections with *I* > 2σ(*I*)
graphite	*R*_int_ = 0.024
φ and ω scans	θ_max_ = 25.0°, θ_min_ = 2.3°
Absorption correction: multi-scan (*SADABS*; Sheldrick, 2003)	*h* = −10→9
*T*_min_ = 0.236, *T*_max_ = 0.965	*k* = −11→16
5082 measured reflections	*l* = −9→10

### Refinement

**Table:**

Refinement on *F*^2^	Secondary atom site location: difference Fourier map
Least-squares matrix: full	Hydrogen site location: inferred from neighbouring sites
*R*[*F*^2^ > 2σ(*F*^2^)] = 0.041	H-atom parameters constrained
*wR*(*F*^2^) = 0.122	*w* = 1/[σ^2^(*F*_o_^2^) + (0.0535*P*)^2^ + 0.2478*P*] where *P* = (*F*_o_^2^ + 2*F*_c_^2^)/3
*S* = 1.04	(Δ/σ)_max_ < 0.001
1827 reflections	Δρ_max_ = 0.16 e Å^−^^3^
131 parameters	Δρ_min_ = −0.18 e Å^−^^3^
0 restraints	Extinction correction: *SHELXTL* (Sheldrick, 2008), Fc^*^=kFc[1+0.001xFc^2^λ^3^/sin(2θ)]^-1/4^
Primary atom site location: structure-invariant direct methods	Extinction coefficient: 0.0080 (18)

### Special details

**Table:**

Geometry. All esds (except the esd in the dihedral angle between two l.s. planes) are estimated using the full covariance matrix. The cell esds are taken into account individually in the estimation of esds in distances, angles and torsion angles; correlations between esds in cell parameters are only used when they are defined by crystal symmetry. An approximate (isotropic) treatment of cell esds is used for estimating esds involving l.s. planes.
Refinement. Refinement of F^2^ against ALL reflections. The weighted R-factor wR and goodness of fit S are based on F^2^, conventional R-factors R are based on F, with F set to zero for negative F^2^. The threshold expression of F^2^ > 2sigma(F^2^) is used only for calculating R-factors(gt) etc. and is not relevant to the choice of reflections for refinement. R-factors based on F^2^ are statistically about twice as large as those based on F, and R- factors based on ALL data will be even larger.

### Fractional atomic coordinates and isotropic or equivalent isotropic displacement parameters (Å^2^)

**Table:**

	*x*	*y*	*z*	*U*_iso_*/*U*_eq_	
O1	0.6568 (2)	0.70399 (12)	0.1437 (3)	0.1020 (7)	
O2	0.21582 (15)	0.66963 (10)	0.42215 (18)	0.0649 (4)	
O3	0.16048 (14)	0.48733 (10)	0.42736 (17)	0.0602 (4)	
C1	0.9079 (2)	0.60873 (18)	0.0925 (3)	0.0775 (7)	
H1A	0.9509	0.6544	0.1740	0.116*	
H1B	0.8682	0.6422	−0.0058	0.116*	
H1C	0.9859	0.5642	0.0773	0.116*	
C2	0.7796 (2)	0.55404 (15)	0.1427 (3)	0.0578 (5)	
H2A	0.7366	0.5081	0.0597	0.069*	
H2B	0.8214	0.5179	0.2392	0.069*	
C3	0.6546 (2)	0.61841 (15)	0.1727 (3)	0.0560 (5)	
C4	0.52728 (19)	0.57857 (13)	0.2399 (2)	0.0463 (5)	
C5	0.4315 (2)	0.64333 (13)	0.2967 (2)	0.0497 (5)	
H5	0.4492	0.7093	0.2916	0.060*	
C6	0.31209 (19)	0.61142 (13)	0.3597 (2)	0.0478 (5)	
C7	0.28263 (19)	0.51162 (14)	0.3649 (2)	0.0478 (5)	
C8	0.3772 (2)	0.44743 (13)	0.3093 (2)	0.0511 (5)	
H8	0.3589	0.3815	0.3125	0.061*	
C9	0.4994 (2)	0.48095 (13)	0.2487 (2)	0.0509 (5)	
H9	0.5636	0.4370	0.2134	0.061*	
C10	0.2320 (3)	0.77064 (14)	0.4052 (3)	0.0708 (7)	
H10A	0.2190	0.7864	0.2940	0.106*	
H10B	0.3322	0.7903	0.4614	0.106*	
H10C	0.1557	0.8036	0.4487	0.106*	
C11	0.1190 (3)	0.38787 (15)	0.4241 (3)	0.0670 (6)	
H11A	0.2027	0.3511	0.4847	0.101*	
H11B	0.0958	0.3654	0.3155	0.101*	
H11C	0.0305	0.3801	0.4697	0.101*	

### Atomic displacement parameters (Å^2^)

**Table:**

	*U*^11^	*U*^22^	*U*^33^	*U*^12^	*U*^13^	*U*^23^
O1	0.0941 (13)	0.0551 (10)	0.182 (2)	0.0115 (8)	0.0862 (13)	0.0284 (11)
O2	0.0603 (8)	0.0511 (8)	0.0924 (11)	0.0016 (7)	0.0363 (8)	−0.0055 (7)
O3	0.0537 (8)	0.0536 (8)	0.0801 (10)	−0.0069 (6)	0.0291 (7)	0.0014 (7)
C1	0.0584 (13)	0.0816 (16)	0.1011 (19)	−0.0035 (12)	0.0364 (13)	−0.0103 (14)
C2	0.0530 (11)	0.0600 (12)	0.0648 (13)	0.0022 (9)	0.0220 (10)	−0.0019 (10)
C3	0.0530 (11)	0.0478 (12)	0.0714 (14)	0.0016 (9)	0.0226 (10)	0.0058 (10)
C4	0.0426 (10)	0.0462 (10)	0.0516 (11)	0.0049 (8)	0.0134 (8)	0.0046 (9)
C5	0.0467 (10)	0.0423 (10)	0.0612 (12)	0.0010 (8)	0.0144 (9)	0.0043 (9)
C6	0.0421 (10)	0.0477 (11)	0.0547 (12)	0.0044 (8)	0.0127 (8)	−0.0013 (9)
C7	0.0425 (10)	0.0509 (11)	0.0511 (11)	−0.0024 (8)	0.0126 (8)	0.0016 (9)
C8	0.0524 (11)	0.0408 (10)	0.0608 (12)	−0.0021 (9)	0.0135 (9)	0.0021 (9)
C9	0.0490 (10)	0.0446 (11)	0.0617 (12)	0.0059 (8)	0.0175 (9)	0.0000 (9)
C10	0.0690 (14)	0.0526 (13)	0.0963 (18)	0.0069 (10)	0.0303 (13)	−0.0095 (11)
C11	0.0650 (13)	0.0597 (13)	0.0828 (16)	−0.0167 (11)	0.0300 (11)	−0.0014 (12)

### Geometric parameters (Å, °)

**Table:**

O1—C3	1.213 (2)	C4—C5	1.398 (2)
O2—C6	1.369 (2)	C5—C6	1.368 (2)
O2—C10	1.418 (2)	C5—H5	0.9300
O3—C7	1.357 (2)	C6—C7	1.410 (3)
O3—C11	1.426 (2)	C7—C8	1.381 (3)
C1—C2	1.511 (3)	C8—C9	1.385 (3)
C1—H1A	0.9600	C8—H8	0.9300
C1—H1B	0.9600	C9—H9	0.9300
C1—H1C	0.9600	C10—H10A	0.9600
C2—C3	1.494 (3)	C10—H10B	0.9600
C2—H2A	0.9700	C10—H10C	0.9600
C2—H2B	0.9700	C11—H11A	0.9600
C3—C4	1.487 (3)	C11—H11B	0.9600
C4—C9	1.381 (2)	C11—H11C	0.9600
			
C6—O2—C10	117.08 (15)	C5—C6—C7	119.73 (16)
C7—O3—C11	117.36 (15)	O2—C6—C7	115.37 (16)
C2—C1—H1A	109.5	O3—C7—C8	125.45 (17)
C2—C1—H1B	109.5	O3—C7—C6	115.27 (16)
H1A—C1—H1B	109.5	C8—C7—C6	119.27 (16)
C2—C1—H1C	109.5	C7—C8—C9	120.20 (17)
H1A—C1—H1C	109.5	C7—C8—H8	119.9
H1B—C1—H1C	109.5	C9—C8—H8	119.9
C3—C2—C1	112.94 (18)	C4—C9—C8	121.00 (17)
C3—C2—H2A	109.0	C4—C9—H9	119.5
C1—C2—H2A	109.0	C8—C9—H9	119.5
C3—C2—H2B	109.0	O2—C10—H10A	109.5
C1—C2—H2B	109.0	O2—C10—H10B	109.5
H2A—C2—H2B	107.8	H10A—C10—H10B	109.5
O1—C3—C4	119.34 (18)	O2—C10—H10C	109.5
O1—C3—C2	120.19 (18)	H10A—C10—H10C	109.5
C4—C3—C2	120.46 (17)	H10B—C10—H10C	109.5
C9—C4—C5	118.62 (16)	O3—C11—H11A	109.5
C9—C4—C3	123.16 (16)	O3—C11—H11B	109.5
C5—C4—C3	118.22 (16)	H11A—C11—H11B	109.5
C6—C5—C4	121.16 (17)	O3—C11—H11C	109.5
C6—C5—H5	119.4	H11A—C11—H11C	109.5
C4—C5—H5	119.4	H11B—C11—H11C	109.5
C5—C6—O2	124.90 (17)		
			
C1—C2—C3—O1	6.0 (3)	C11—O3—C7—C8	−5.2 (3)
C1—C2—C3—C4	−173.37 (19)	C11—O3—C7—C6	175.36 (16)
O1—C3—C4—C9	167.5 (2)	C5—C6—C7—O3	−179.08 (15)
C2—C3—C4—C9	−13.1 (3)	O2—C6—C7—O3	1.6 (2)
O1—C3—C4—C5	−12.5 (3)	C5—C6—C7—C8	1.4 (3)
C2—C3—C4—C5	166.88 (18)	O2—C6—C7—C8	−177.89 (16)
C9—C4—C5—C6	−0.1 (3)	O3—C7—C8—C9	−179.64 (17)
C3—C4—C5—C6	179.88 (18)	C6—C7—C8—C9	−0.2 (3)
C4—C5—C6—O2	177.98 (17)	C5—C4—C9—C8	1.4 (3)
C4—C5—C6—C7	−1.3 (3)	C3—C4—C9—C8	−178.62 (18)
C10—O2—C6—C5	6.7 (3)	C7—C8—C9—C4	−1.2 (3)
C10—O2—C6—C7	−174.01 (17)		

### Hydrogen-bond geometry (Å, °)

**Table:**

*D*—H···*A*	*D*—H	H···*A*	*D*···*A*	*D*—H···*A*
C11—H11C···O2^i^	0.96	2.66	3.607 (2)	171.
C8—H8···O1^ii^	0.93	2.50	3.419 (3)	171.

Symmetry codes: (i) −*x*, −*y*+1, −*z*+1; (ii) −*x*+1, *y*−1/2, −*z*+1/2.
